# Cell-Free DNA Kinetics in a Pre-Clinical Model of Head and Neck Cancer

**DOI:** 10.1038/s41598-017-17079-6

**Published:** 2017-12-01

**Authors:** Nidal Muhanna, Marco A. Di Grappa, Harley H. L. Chan, Tahsin Khan, Cheng S. Jin, Yangqiao Zheng, Jonathan C. Irish, Scott V. Bratman

**Affiliations:** 1 Princess Margaret Cancer Centre, University Health Network, Toronto, ON Canada; 2 Department of Otolaryngology – Head & Neck Surgery, University of Toronto, Toronto, ON Canada; 3 Department of Radiation Oncology, University of Toronto, Toronto, ON Canada

## Abstract

In cancer patients, circulating tumour-derived DNA (ctDNA) levels imperfectly reflect disease burden apparent on medical imaging. Further evaluation of ctDNA levels over time is needed to better understand the correlation with tumour growth and therapeutic response. We describe ctDNA kinetics within an orthotopic, immunocompetent preclinical rabbit model of local-regionally advanced head and neck squamous cell carcinoma (HNSCC). Monitoring primary tumour and metastatic lymph node volume by computed tomography (CT), we observed a correlation between ctDNA levels and tumour burden. We found that ctDNA detection could precede evidence of tumour on CT. Sensitivity and specificity of ctDNA detection in this model was 90.2% (95% C.I.: 76.9–97.3%) and 85.7% (95% C.I.: 67.3–96.0%), respectively. Rapid tumour growth followed by auto-necrosis and tumour volume contraction produced a spike in ctDNA levels, suggesting that viable tumour cells may be required for sustained ctDNA release. Following surgical resection, both ctDNA and total plasma DNA were correlated with recurrent tumour volume. Our results reveal the complex kinetic behaviour of ctDNA and total plasma DNA upon tumour growth or surgery. This pre-clinical model could be useful for future studies focused on elucidating mechanisms of ctDNA release into the circulation from primary and metastatic sites.

## Introduction

Recent refinements to diagnostic practices and prognostic classifiers of cancer have exploited tumour-specific molecular characteristics. Unlike tissue-based biomarkers that often necessitate invasive procedures and cannot easily be serially assessed, blood-based biomarkers have the potential to reflect tumour burden and provide insight into longitudinal tumour behaviour in a convenient and timely manner. Cancer is characterized by somatic genetic aberrations^1^, and tumour-specific genomic features can be utilized to distinguish tumour-derived DNA within the circulation^2^. Cell-free plasma DNA is composed of predominantly short (~140–200 bp) DNA fragments, and is derived from both normal and malignant tissues. Circulating tumour-derived DNA (ctDNA) is shed into the bloodstream following cell death, and levels are related to tumour stage and prognosis^2–6^. Potential clinical applications of ctDNA analysis include screening, prognostication, monitoring of disease burden, evaluation of treatment response, minimal residual disease detection, and early detection of recurrence^7^.

ctDNA levels change over time and in response to treatment. An increase of ctDNA level has frequently been found to correlate with higher overall disease burden in multiple cancer types, such as melanoma^8,9^, ovarian^10^, breast^10^, colon^4^ and prostate cancer^2,11^. In nasopharyngeal cancer patients for example, plasma Epstein–Barr virus DNA levels fall precipitously following surgery or radiotherapy and frequently rise again months prior to clinical detection of recurrence^12–14^. Moreover, a recent analysis of CA 15-3 (a plasma protein biomarker), circulating tumour cells, and ctDNA in patients with advanced breast cancer revealed that ctDNA served as the most rapid indicator of treatment response^15^. A more complete understanding of ctDNA kinetics has nonetheless been hampered by inherent challenges with serial phlebotomy of cancer patients receiving therapy. Pre-clinical animal models can provide an alternative system for assessing ctDNA kinetics^16,17^; however, to date these models have relied on subcutaneous tumour injections into immunocompromised hosts. Such non-physiologic conditions potentially limit the generalizability of findings from these studies. Thus, there is a need to develop new preclinical models for the analysis of ctDNA kinetics.

Head and neck cancer is the sixth most common cancer type worldwide^18^. Head and neck squamous cell carcinoma (HNSCC) classically has carried a poor overall prognosis; however, patients with human papillomavirus (HPV)-related oropharyngeal cancer, which has increased in incidence over the past 3 decades^19^, are now recognized to have higher survival rates than their HPV-negative counterparts^20,21^. Still, outcomes for HPV-related HNSCC patients remain heterogeneous, so further risk stratification strategies are warranted. Recent studies have demonstrated that HPV DNA is detectable in the majority of patients prior to treatment and declines during and after treatment^22–24^. From this prior work, ctDNA shows great promise as a biomarker that could improve the precision of risk stratification and identify patients with residual or recurrent disease after treatment.

There are currently few suitable pre-clinical animal models for HPV-related HNSCC^25^, and plasma HPV DNA kinetics in animal models have not been reported. Here, we examine ctDNA kinetics in an orthotopic, immunocompetent rabbit model of papillomavirus-associated squamous cell carcinoma. The VX2 tumour derived from the domestic rabbit is associated with the cottontail rabbit papillomavirus (CRPV), which like HPV participates in papillomatosis and carcinogenesis of infected squamous epithelium^26,27^. The transplantable VX2 carcinoma can be serially propagated in rabbits and used to model various cancer types including HNSCC^28–31^. After being injected into the buccal mucosa, VX2 tumour cells metastasize to regional lymph nodes, thus mimicking the natural history of HPV-related HNSCC in humans. This study provides insights into the mechanisms of ctDNA release into the circulation from primary and metastatic sites over time and in response to surgical therapy.

## Results

### VX2 tumour model of papillomavirus-associated HNSCC

**Figure 1:**
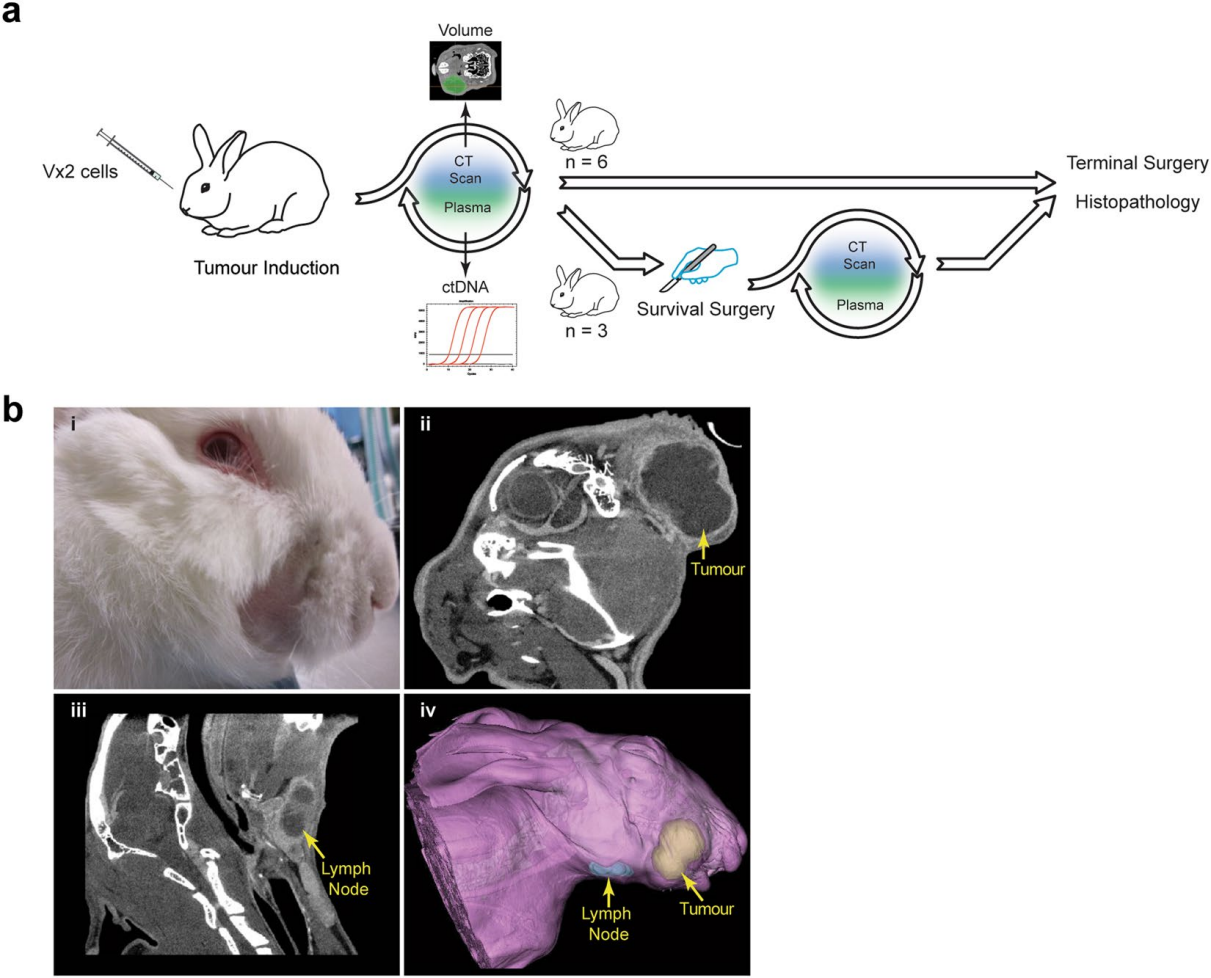
Overview of the rabbit VX2 HNSCC model. (**a**) Schematic depicting experimental design. Nine rabbits with VX2 cells injected into the buccal mucosa underwent serial blood collection and CT imaging. The primary tumours of three of these rabbits were resected and monitored for recurrence. (**b**) Representative images of gross primary tumour (i), and sagittal CT images of primary tumour (ii), and lymph node (iii) along with a 3D surface rendering of CT data showing both of the above (iv).

The VX2 rabbit tumour line has previously been used as an HNSCC model^28–31^. We first confirmed that following tumour injection, tumours would form and that metastases could be detected within regional lymph nodes (Fig. 1). VX2 tumour cells were injected into the right buccinator muscle, and each rabbit (n = 9) was monitored by physical palpation and CT of the head and neck region coinciding with phlebotomy and plasma isolation twice a week (Fig. 1a). Survival surgery was performed on three animals (Rabbit 6, 7 and 8), and terminal surgery and histopathology was performed on all animals. While metastases were readily detected within the mandibularis caudalis lymph node, distant metastases to other organs were not observed at the time of terminal surgery (data not shown). To determine disease burden over time, the primary buccal tumour and mandibularis caudalis lymph node were contoured volumetrically on each CT image set (Fig. 1b).

### Growth kinetics and lymph node metastasis of tumour-bearing subjects

**Figure 2:**
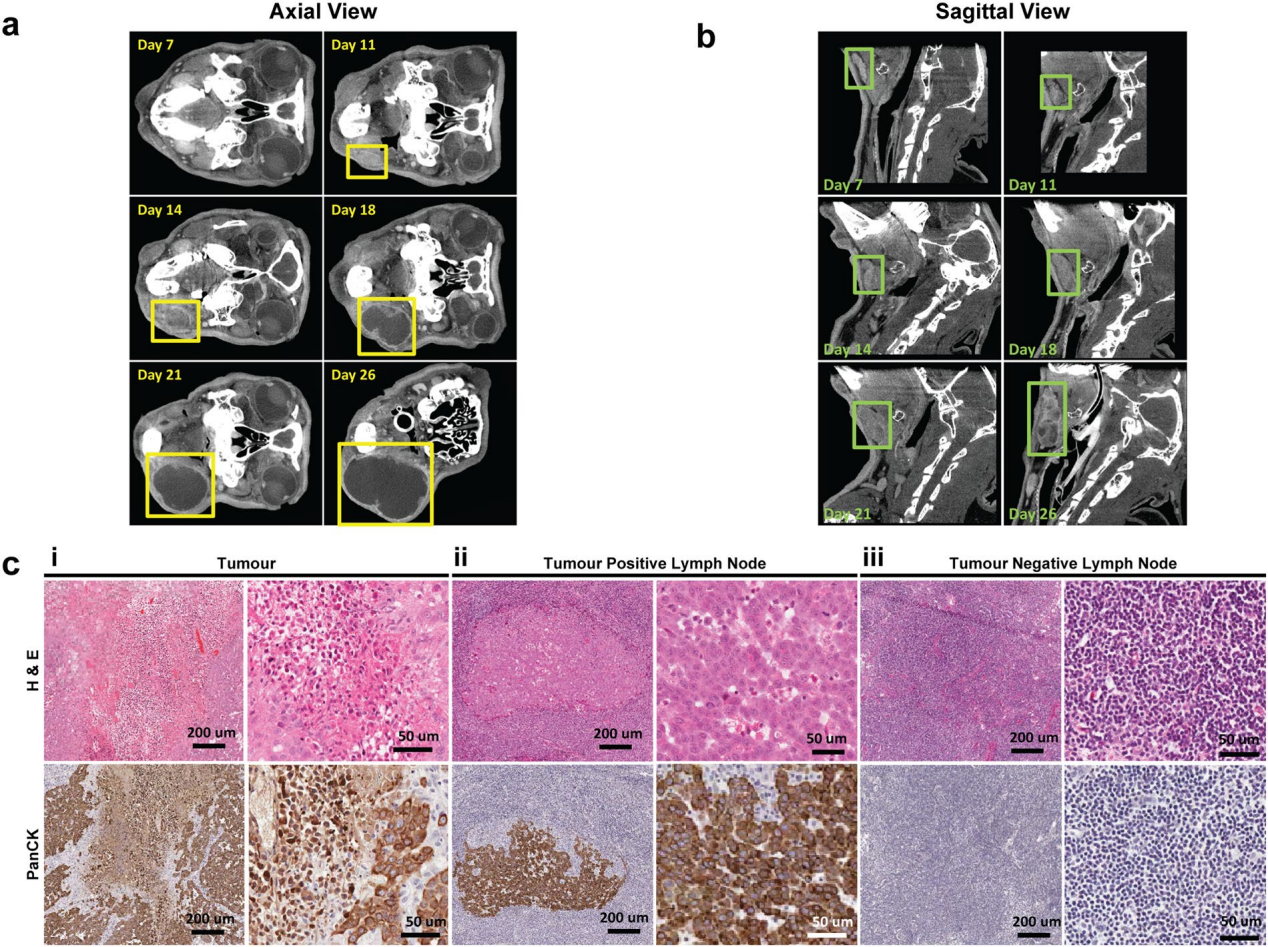
Characterization of the rabbit VX2 HNSCC model. Axial CT images showing the growth of the primary tumour (**a**) and sagittal CT images showing the growth of a lymph node (**b**) of Rabbit 3. (**c**) Representative histology H & E (top row) and pan-cytokeratin (bottom row) staining of primary tumour (i), tumour-positive (ii) and -negative (iii) sections of lymph node. Scale bars: 200 µm; 50 µm.

We analyzed the pattern of tumour growth and lymphatic spread of the VX2 HNSCC animal model. Tumour and lymph node volumes were determined at each time point for all nine VX2 tumour injected rabbits (Fig. 2a and b). Eight rabbits developed detectable tumours at the primary injection site within 6–14 days (Supplementary Figure 1). Only Rabbit 9 did not develop a detectable tumour at the injection site. Primary tumours eventually exhibited exponential growth, reaching a minimum volume of 722.32 mm^3^ (Rabbit 6) and a maximum volume of 17,945.43 mm^3^ (Rabbit 3) prior to surgery. The mandibularis caudalis lymph node that was closest in proximity to the primary tumour also exhibited growth in tumour-bearing animals (Rabbits 1–8). As expected, the lymph node did not exhibit growth in the animal lacking a primary tumour (Rabbit 9).

Although the primary tumour in each animal initially appeared as a solid mass on CT, most tumours eventually developed central hypodensity, indicating auto-necrosis. For example, the primary tumour in Rabbit 3 was detectable by day 11 and became auto-necrotic from day 18 onwards (Fig. 2a). Similarly, the lymph nodes in tumour-bearing rabbits also exhibited visible growth based on CT, followed by subsequent appearance of central hypodensities (Fig. 2b). Central tumour auto-necrosis was confirmed on final pathology by H&E staining (Fig. 2c).

Terminal surgery was eventually performed on all rabbits, at which point, the tumours and metastatic lymph nodes were resected to confirm malignancy in conjunction with histological analyses (Fig. 2c). Staining with H&E and pan-cytokeratin confirmed the presence of metastatic tumour deposits within lymph nodes of all tumour-bearing rabbits. As expected, the lymph node of Rabbit 9 did not exhibit malignant histopathology. Thus, the pattern of metastatic spread in this animal model closely resembles that of human HNSCC.

### Detection and quantification of CRPV DNA from VX2 tumour cells

**Figure 3:**
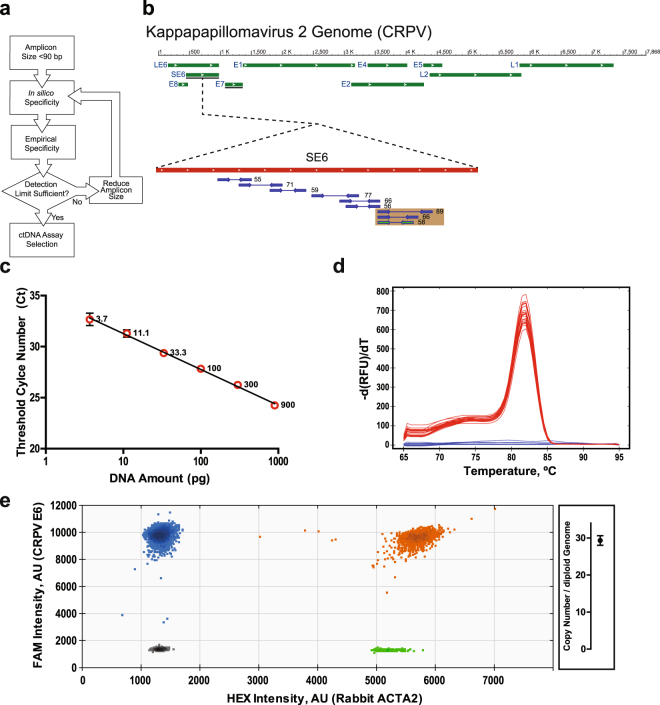
Detection and quantification of CRPV DNA in VX2 cells. (**a**) Flow diagram of the iterative process of optimizing and selecting a sufficiently specific and sensitive qPCR assay for detection of CRPV DNA. (**b**) Map of the CRPV genome highlighting the E6 open reading frame and the sets of qPCR primers tested during optimization process referred to in (**a**). Beige boxes indicate sets of primers with the same 5′ start site but differing in amplicon size. (**c**) Plot of qPCR cycle threshold (Ct) *vs*. amount of VX2 DNA for CRPV E6 qPCR assay. (**d**) Melt curve for CRPV E6 qPCR assay (red) showing no signal in uninjected rabbit plasma (blue) (n = 4 rabbits). (**e**) Quantification of copy number of CRPV genome in VX2 rabbit tumour line by multiplexed ddPCR. 2-D plot of droplet fluorescence for FAM and HEX fluorophores from probes for CRPV E6 gene and rabbit ACTA2 probes respectively. The CRPV copy number per diploid genome: 29.3 ± 1.3. Error bars represent SD.

To detect ctDNA derived from VX2 cells within rabbit plasma, we developed a qPCR assay for use on cell-free plasma DNA. We utilized an iterative process to design and validate specific qPCR assays that would identify CRPV sequences within small DNA fragments (Fig. 3a). The *E6* open reading frame (ORF) was selected for candidate assays (Fig. 3b) due to its known amplification within VX2 genomic DNA^32^ and its well-characterized role in oncogenesis. A 58-bp *E6* assay demonstrated robust detection of CRPV sequences from as little as 3.7 pg VX2 genomic DNA (Fig. 3c) without any signal from uninjected rabbit plasma (Fig. 3d). This optimized assay was selected for use in subsequent analyses. The copy number per diploid genome of the CRPV genome in the VX2 rabbit tumour line was determined to be 29.3 ± 1.3 by multiplexed droplet digital PCR (ddPCR) (Fig. 3e).

### Plasma CRPV DNA levels are correlated with tumour burden in the VX2 HNSCC model

**Figure 4:**
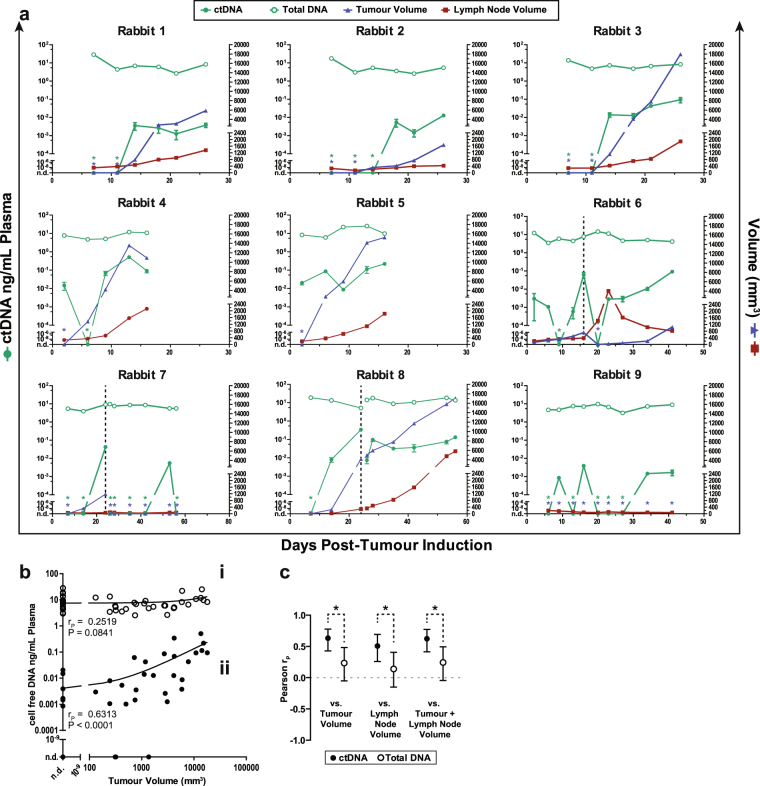
Plasma ctDNA and total plasma DNA correlation with tumour and lymph node volume. (**a**) Summary of tumour volume (blue), lymph node volume (red), plasma ctDNA concentration (green closed), and total DNA concentration (green open) for all rabbits in the study (n = 9). Vertical dotted line (black) represents the day of tumour resection surgery for Rabbits 6, 7, and 8. Asterisks at specific time points indicate a value of 0 corresponding to the color of the data point (i.e. blue asterisk at day 3 = tumour volume is 0 mm^3^). Error bars represent SD. n.d. denotes not detectable. (**b**) Scatter plots showing correlation between ctDNA and tumour volume (filled circles, ii) and between total plasma DNA and tumour volume (open circles, i) at all pre-operative time points for all rabbits in the study (n = 9 rabbits; n = 48 total individual timepoints). Linear regression trend lines are shown. (**c**) Correlation (Pearson r, mean ± 95% confidence interval) between plasma ctDNA or total plasma DNA and the indicated volume parameter for pre-operative timepoints for all rabbits. Asterisks indicate P < 0.05.

We measured ctDNA levels within rabbit plasma using the optimized CRPV *E6* ORF qPCR assay as well as total cell-free plasma DNA using a rabbit *LINE-1* qPCR assay. As ctDNA analysis coincided with volumetric tumour measurements by CT, we were able to determine the kinetics of ctDNA in relation to tumour burden. Initial detection of ctDNA preceded evidence of tumour on CT in 3 of 8 animals (Fig. 4a). Sensitivity and specificity of ctDNA detection was 90.2% (95% C.I.: 76.9–97.3%) and 85.7% (95% C.I.: 67.3–96.0%), respectively (Supplementary Tables 1 and 2). Levels of ctDNA strongly correlated with volumetric tumour measurements (Fig. 4b and c). There was no significant correlation between total plasma DNA levels and tumour volume (Fig. 4b and c).

Primary tumours were generally larger than metastatic lymph nodes, so we expected that ctDNA levels would more closely reflect primary tumour volumes. In the pre-operative setting, although the correlation of ctDNA levels with primary tumour volume (R = 0.63, P < 0.0001; Fig. 4c and Supplementary Figure 2A) was numerically higher than with lymph node volume (R = 0.51, P = 0.0002; Fig. 4c and Supplementary Figure 2B) or with total volume of tumour and lymph node (R = 0.62, P < 0.0001; Fig. 4c and Supplementary Figure 2C), the difference was not statistically significant, possibly due to highly correlated growth of the primary tumour and lymph node in this model (Fig. 4a). Taken together, these results indicate that levels of tumour-specific plasma DNA is reflective of tumour burden in this HNSCC animal model.

### Impact of tumour auto-necrosis on ctDNA levels

**Figure 5:**
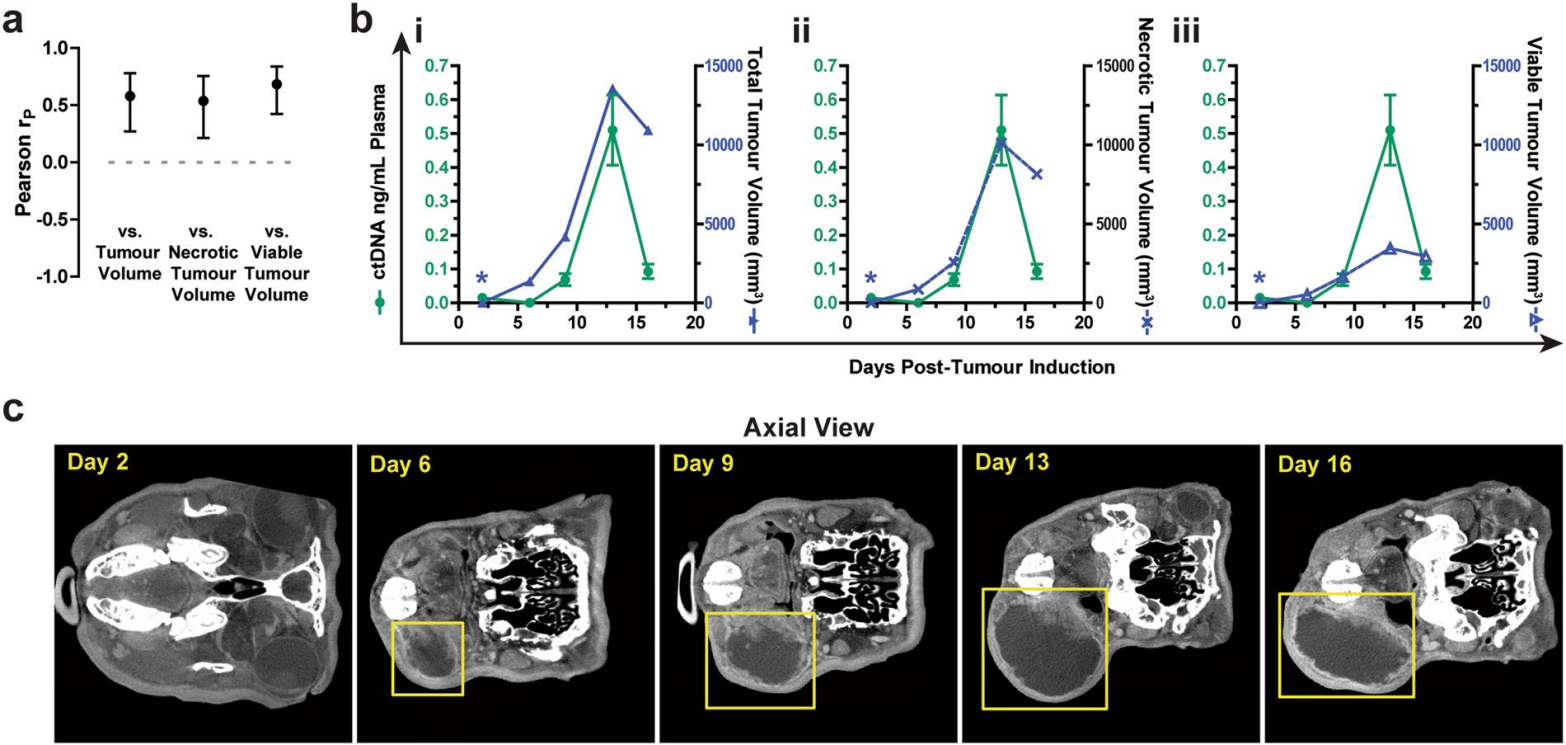
Effect of necrosis on plasma ctDNA levels. (**a**) Correlation (Pearson r, mean ± 95% confidence interval) between plasma ctDNA and the indicated volume parameter for pre-operative timepoints in which tumour was visible by CT in all rabbits (n = 8 rabbits; n = 29 total individual timepoints). (**b**) Representative CT images demonstrating Rabbit 4 primary VX2 tumour growth. Yellow boxes highlight region of tumour growth. (**c**) Relationship between plasma ctDNA and total tumour volume (i), necrotic tumour volume (ii) and viable/solid tumour volume (iii) for Rabbit 4. Blue asterisks denote non-detectable tumour size. Error bars for ctDNA values represent SD.

We next examined the relationship between tumour auto-necrosis and ctDNA kinetics. Enlarging tumours developed necrotic cores that presented as central hypodensities on CT (Fig. 2). CT values (Hounsfield Units, HU), which are lower in areas of tumour necrosis than in viable tumour, demonstrated an inverse relationship with the total tumour volume and with ctDNA levels (Supplementary Figure 3). Across the entire study, when the necrotic and viable tumour regions were contoured separately, each was correlated to a similar degree with ctDNA levels (Fig. 5A), and each was also highly correlated to one another (data not shown).

In one instance (Rabbit 4) the tumour volume contracted after an initial phase of rapid growth (Fig. 5b and c). ctDNA levels increased initially with tumour growth and then fell dramatically when the tumour volume declined. The necrotic tumour volume appeared to reflect more closely ctDNA levels during the phase of rapid tumour growth; however, upon tumour contraction the necrotic tumour volume provided an overestimate of ctDNA levels. This case study illustrates the complex temporal relationship that can exist between tumour growth, necrosis, and ctDNA release.

### ctDNA kinetics following surgical removal of primary tumour

In order to assess ctDNA kinetics following primary tumour resection, once tumours formed we performed survival surgery on Rabbits 6, 7 and 8. In each case, ctDNA was readily detectable immediately prior to surgery (Fig. 4a). We observed three distinct outcomes for each rabbit: recurrence following complete resection of the primary tumour (Rabbit 6), no recurrence following complete resection of the primary tumour (Rabbit 7), and recurrence following incomplete resection of the primary tumour (Rabbit 8). For Rabbit 6, recurrent tumour growth was observed 18 days post-surgery (day 34 post-tumour induction). For Rabbit 8, gross total resection was achieved, but positive margins were detected; post-operative CT showed abnormal soft tissue followed by clear local recurrence.

**Figure 6:**
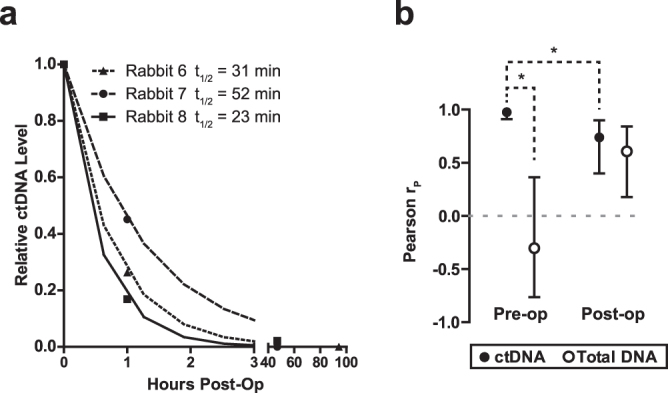
Plasma ctDNA levels following surgery. (**a**) Relative ctDNA level post-operation for rabbits having undergone surgery (n = 3 rabbits). The estimated value for the half-life of ctDNA in plasma, based on one-phase decay, is shown for each curve. (**b**) Correlation (Pearson r, mean ± 95% confidence interval) between ctDNA or total plasma DNA vs. tumour + lymph node volume from pre- and post-surgical removal of the tumour for only those rabbits that underwent surgery (Rabbits 6, 7 and 8). Pearson r (r_p_) and P values are indicated. Dashed lines represent 95% confidence interval. Asterisks indicate P < 0.05.

Following surgery, ctDNA levels fell with a half-life of 23–52 min (Fig. 6a). ctDNA became undetectable for Rabbits 6 and 7 but remained in the detectable range for Rabbit 8 despite gross total resection. ctDNA was detectable at all post-operative time points for which recurrent tumour was seen by CT (Fig. 4a). ctDNA levels continued to be correlated with tumour volume, albeit less strongly than in the pre-operative setting (Fig. 6b). Unexpectedly, although total plasma DNA levels were not correlated with tumour volume prior to surgery (Fig. 4), like ctDNA, total plasma DNA levels were also correlated with tumour volume in the post-operative setting (Fig. 6b).

**Figure 7:**
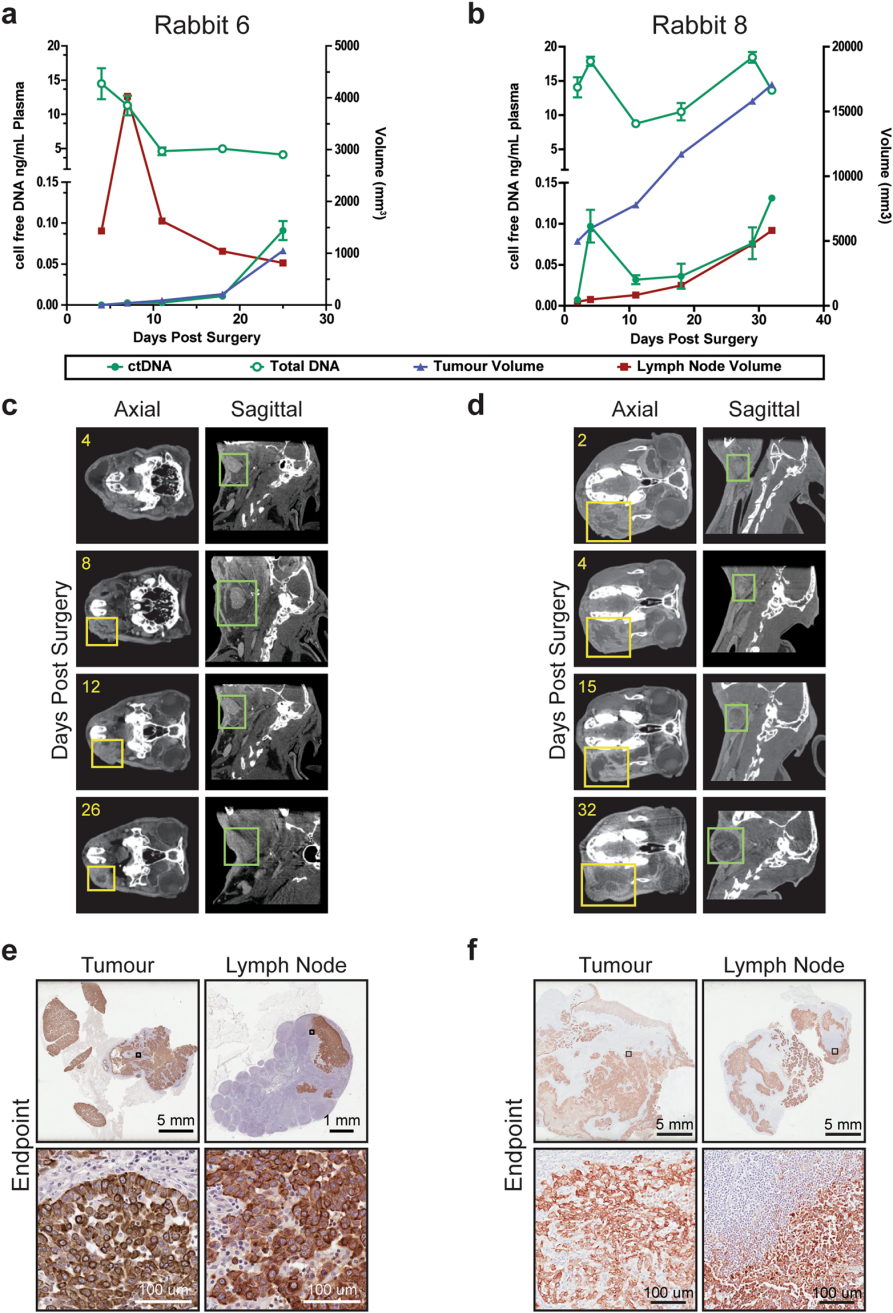
Plasma ctDNA levels in the setting of tumour recurrence and lymph node swelling. Data from Rabbit 6 (**a**,**c**,**e**) and Rabbit 8 (**b,d,f**) are shown. (**a,b**) Plots showing primary tumour (blue) and lymph node (red) volumes, as well as the plasma ctDNA (full green circles) and total plasma DNA (open green circles) concentrations at the indicated post-operative days. (**c,d**) Axial and sagittal orientation serial CT images of primary tumour (yellow boxes) and lymph node (green boxes) over time course at the indicated post-operative days. (**e,f**) Low- and high-magnification histology images of pan-cytokeratin staining for recurrent primary tumour and lymph node tissue from endpoint dissection.

To investigate the weaker correlation between ctDNA and tumour volume observed in the post-operative compared to the pre-operative setting, we performed histopathologic analysis of resected tissues at end point (Fig. 7). For Rabbit 8, residual tumour was detected by CT after surgery, and ctDNA kinetics at later time points reflected growth at both the primary site and lymph node. Pan-cytokeratin staining of end point tissues demonstrated extensive tumour involvement at both sites. In contrast, for Rabbit 6, the lymph node volume dramatically increased following surgery and then gradually subsided while ctDNA displayed a continuous rise. At end point, the lymph node tissue demonstrated only a small focus of tumour staining positive for pan-cytokeratin, indicating that the swelling seen on CT reflected post-operative edema as opposed to tumour growth. Thus, we can infer that for Rabbit 6 the primary tumour was the principal source of ctDNA, whereas for Rabbit 8 both the primary tumour and the lymph node metastasis likely contributed.

## Discussion

Detection of ctDNA has emerged as a promising strategy for tracking tumour burden and assessing response to treatment, yet few studies have been performed to characterize the kinetics of ctDNA release in tumour-bearing subjects with local-regional disease. For this purpose, we have adopted the VX2 rabbit model of papillomavirus-associated HNSCC, which phenotypically mimics the human disease counterpart in terms of its metastatic lymph node spread in an immunocompetent host. We developed a robust qPCR assay with high sensitivity and specificity for papillomavirus DNA derived from VX2 tumour cells. This assay is based on detection of the viral *E6* ORF, which is similar to published ctDNA detection methods for HPV-associated malignancies^22–24^.

Using this model, we observed a correlation between tumour volume and ctDNA originating from the injected VX2 cells. ctDNA detection displayed high sensitivity and specificity for tumour growth detected by CT. In the pre-operative setting, the strongest correlation was observed between primary tumour volume and ctDNA, whereas little-to-no correlation was observed between tumour volume and total plasma DNA, which includes DNA from normal non-malignant cells such as peripheral blood leukocytes. Although we also observed correlation between ctDNA levels and lymph node growth, this correlation was numerically weaker and could partially be explained by coincident tumour growth at the primary site.

One rabbit displayed intermittently detectable ctDNA despite lack of any detectable tumour growth during the observation period. It is possible that a tumour could have arisen in this animal with additional observation time or that a small tumour could have been present below the detection threshold of CT. We also speculate that the ctDNA signal could have emanated from the injected cells that may have died slowly, leading to continuous release of low levels of ctDNA into the bloodstream. This observation, if confirmed in future studies, could have implications for settings of tumour cell dormancy or other scenarios involving slow cellular turnover where the utility of ctDNA analysis has not been firmly established.

This model can produce tumours that grow rapidly and undergo auto-necrosis. As tumours grew, we found that the rise in ctDNA levels closely resembled that of the necrotic portion of the tumour. However, in one instance, when auto-necrosis became so profound that total tumour size contracted, ctDNA levels plummeted. This case study suggests that the release of ctDNA from necrotic or necrosing tumour is time-dependent and may require continual tumour growth to fuel ctDNA release. These findings, if confirmed in larger studies, could have important implications for the use of ctDNA to monitor response to treatment, as many cancer therapies cause tumour necrosis and would thus be expected to produce a temporary spike in ctDNA levels. Our results should be interpreted with caution, however, given the small number of animals included in this study and the fact that human tumours generally display much slower growth trajectories which might affect ctDNA kinetics. The relative contribution of necrosis, apoptosis, and other modes of cell death to ctDNA levels and kinetics could be explored further with this model in future studies.

In the post-operative setting, both ctDNA and total plasma DNA were weakly correlated with tumour volume. We speculate that this could be attributed to inflammatory changes in the operative bed that interfere with accurate measurement of recurrent tumour while producing DNA release from non-cancerous tissues into the circulation. These findings point to the difficulties with interpretation of post-operative imaging and the potential value of ctDNA analysis for distinguishing swelling/inflammation from recurrent disease.

There may be challenges in translating results of our work into the clinical realm. First, as a proportion of total blood volume the 3 mL blood draw from rabbits utilized in our study is equivalent to ~70 mL blood taken from a human subject, which would not be practical for serial assessments. Second, multiple HPV subtypes cause human cancers, including HPV-16, -18, -31, -33, -35, and others^33^, so upfront genotyping of the tumour may be necessary, or else a strategy must be employed that can simultaneously detect DNA from multiple HPV subtypes while maintaining exquisite sensitivity and specificity. Third, we note that the CRPV genome is amplified ~30-fold in VX2 cells, which exceeds the median number of integrants of HPV genomes in human cancer^34^. Thus, an *E6* qPCR assay may be less sensitive for detection of ctDNA in the majority of patients with HPV-driven cancer. With further technological advances, however, we envision that HPV-based ctDNA detection will one day become routine in clinical practice.

In conclusion, this VX2 orthotopic model of papillomavirus-related HNSCC has provided new insights into the release of cell-free DNA into the circulation from primary tumours, metastatic lymph nodes, and normal tissues. Further characterization of ctDNA kinetics in animal models and in patient samples is warranted in order to determine its clinical suitability as a biomarker for HNSCC.

## Materials and Methods

### Tumour cell line propagation

VX2 tumours were propagated by injecting 500 μL of suspended VX2 cells (5 × 10^6^/mL) into the quadriceps of New Zealand white rabbits, and tumours were harvested after approximately 3 weeks. Harvested tumours were stored in liquid nitrogen prior to tumour induction. Prior to tumour induction in rabbits, the tumour pieces were thawed and cut using a sterile scalpel and subsequently placed onto a 70 μm cell strainer sitting on a 50 mL tube (Beckton Dickinson). A syringe plunger was used to mince the cells and ~500 μL HBSS was used to suspend the cells in the strainer to reach the concentration of 5 × 10^6^ cells/mL.

### Animal model

Experiments were performed using male New Zealand white rabbits weighing 2.5–3.0 kg (Charles River, Wilmington, Massachusetts). Male rabbits were injected with 300 μL of a high-density (approximately 5 × 10^6^/mL) VX2 cell suspension into the cheek muscles (buccinator). Tumour development and lymph node metastases were monitored using computed tomography (CT) twice a week. The experimental protocol was approved by the Animal Care Committee (ACC) of the University Health Network, and all experiments were performed in accordance with the guidelines and regulations of the ACC.

### MicroCT imaging

All rabbits underwent serial imaging with MicroCT (Locus Ultra, GE Medical Systems, Milwaukee, WI, USA). Under anesthesia, rabbits were placed in ventral recumbency on the scanning bed and injected intravenously with 10 mL Omnipaque (GE Health Care, USA) at 0.3 mg/kg administered over one minute following 2 mL 0.2% (2 mg/mL) heparin sodium salt. MicroCT scans were acquired at 80kvp and 50 mA, image reconstruction using traditional filter back projection with graphic processing unit (GPU) improving image reconstruction speed, image resolution 0.154 mm × 0.154 mm pixel size and slice thickness in 0.154 mm.

### CT image analysis

All CT-based image analysis was performed using Microview (GE Healthcare, Milwaukee, WI, USA) and a custom in-house program written using MATLAB (MathWorks®, Natick, Massachusetts). The mean and standard deviation of the voxel signal distribution within each VOI was calculated.

### Tumour and lymph node volume contouring

Volumes of tumours and the mandibularis caudalis lymph node on the ipsilateral side in each subject were manually contoured using Mimics 16.0 (x64), including the total tumour or lymph node volume, the hypodense non-ehnancing (non-viable) tumour or lymph node volume, and enhancing (viable) tumour or lymph node volume.

### Blood collection for circulating DNA analysis

For all rabbits in the study, 3 mL of blood was collected into EDTA tubes (Beckton Dickinson) at serial time points following tumour induction. Blood samples were collected 5–10 minutes prior to microCT imaging. The length of time that the rabbits were monitored was dictated by the size of the respective tumours and the health status of those rabbits in accordance with the University Health Network Animal Care Committee policies. Within 1 hour of blood draw, plasma was separated from the blood cell pellet by centrifugation at 2500 x g for 10 minutes. A second spin at 16,100 x g for 10 minutes was performed on the separated plasma, and the supernatant was then aliquoted in DNA/RNA LoBind microcentrifuge tubes (Eppendorf) and stored at −80 °C.

### Circulating DNA isolation and quantification

Total plasma DNA was purified from all available separated plasma (1–2 mL per time point) using the QIAamp Circulating Nucleic Acid Kit (Qiagen) without the use of carrier RNA. DNA was eluted into 100 µL AVE buffer and then concentrated using Agencourt AMPure beads (Beckman Coulter) into 22 µL TE buffer (10 mM Tris-HCl pH 8.0, 0.1 mM EDTA) for downstream quantitative PCR (qPCR) analysis. Total plasma DNA concentration was determined by qPCR using an assay detecting the rabbit *LINE-1* repeat element (forward 5′-TCAGGAAACCCCAGAAAGTATGC; reverse 5′-TTTGATTTCTTGAATGACCCAGTGT) and a standard curve constructed from purified genomic DNA from VX2 cells. Quadruplicate qPCR reactions were carried out in 10 microliters each, consisting of SsoAdvanced Universal SYBR Green Supermix (Bio-Rad) or PowerUP SYBR Green Mastermix (Life Technologies) and 250 nM oligos. Of the 22 µL purified plasma DNA, 4 µL of a 1:10 dilution was used as input into each qPCR replicate reaction. Forty cycles of two-step thermocycling and SYBR Green detection were performed using a CFX384 Touch Real-Time PCR Detection System (Bio-Rad).

### Quantification of VX2 ctDNA

The concentration of VX2-derived DNA within plasma DNA was determined by qPCR. Sequences specific for the Kappapapillomavirus 2 (CRPV) *E6* ORF were selected for PCR primer design using Primer3 (http://primer3plus.com/). Default parameters were used with the exception of maximum product size, which was set at 90 bp. The selected assay amplified a 58 bp fragment within *E6* (forward 5′-GATCCTGGACCCAACCAGTG; reverse 5′-CTGTTCCGGACGGATAGCTG). A standard curve was constructed from purified genomic DNA from VX2 cells. Quadruplicate qPCR reactions were carried out in 10 microliters each, consisting of SsoAdvanced Universal SYBR Green Supermix (Bio-Rad) or PowerUP SYBR Green Mastermix (Life Technologies) and 250 nM oligos. Of the 22 µL purified plasma DNA, 4 µL was used as input into each qPCR replicate reaction.

### Survival surgery

Surgery was performed at day 16 after tumour induction. Tumour volume of 1500 mm^3^ was chosen in order to ensure that the tumour resection would not affect critical structures and vital functions of the rabbits. The primary tumour was dissected and sent for pathology and immunostaining analysis.

### Histopathological evaluation

Resected tissues were fixed in formalin, embedded in paraffin blocks, cut at the thickness of 8 μm and stained with hematoxylin and eosin (H&E) and pan-cytokeratin (AE1/AE3) using adjacent slides. All histopathology images were analyzed using ImageScope after scanning.

### Statistical analysis

Statistical tests and determinations of 95% confidence intervals were performed in GraphPad Prism and R. Correlation was determined using the Pearson test. For calculating sensitivity and specificity, a true positive result was defined as detectable ctDNA when tumour was visible on CT at the same or subsequent time point, a false negative result was defined as undetectable ctDNA when tumour was visible on CT at the same or subsequent time point, a false positive result was defined as detectable ctDNA in pre-injected rabbit plasma or in Rabbit 7 follow surgery (due to lack of tumour recurrence), and a true negative result was defined as undetectable ctDNA in pre-injected rabbit plasma or in Rabbit 7 following surgery. All error bars represent standard deviations. P-values of <0.05 were considered significant. All P-values were two-sided. The datasets generated during and/or analysed during the current study are available from the corresponding author on reasonable request.

## Electronic supplementary material

Supplementary Information
