# Supplementary material for: Cell-Free DNA Kinetics in a Pre-Clinical Model of Head and Neck Cancer
Source: Sci Rep. 2017 Dec 1;7:16723. doi: 10.1038/s41598-017-17079-6 (PMC5711859; doi:10.1038/s41598-017-17079-6)
Supplement: Supplementary file 1 — Supplementary Information [file 41598_2017_17079_MOESM1_ESM.pdf]

Supplementary Information

Cell-Free DNA Kinetics in a Pre-Clinical Model of Head and Neck Cancer

Nidal Muhanna, MD PhD[1,2], Marco A. Di Grappa[1], Harley Chan, PhD[1], Tahsin Khan[1], Cheng S. Jin, PhD[1], Yangqiao Zheng[1], Jonathan C. Irish, MD[1,2], and Scott V. Bratman, MD PhD[1,3,*]

[1] Princess Margaret Cancer Centre, University Health Network, Toronto, ON, Canada.
[2] Department of Otolaryngology – Head & Neck Surgery, University of Toronto, Toronto, ON, Canada
[3] Department of Radiation Oncology, University of Toronto, Toronto, ON, Canada.

[*] **Corresponding author:**

Scott V. Bratman
101 College Street
MaRS/PMCRT 14-313
Toronto, Ontario M5G1L7
Canada
Tel: 647-634-7077
Fax: 416-946-6561
Email: scott.bratman@rmp.uhn.ca

Supplementary Table 1 Muhanna *et al.*

Table S1. Contingency table for ctDNA assay

	Positive	Negative	Total
	True Positive	*False Positive*	
**Positive**	37	4	41
	False Negative	*True Negative*	
**Negative**	4	24	28
**Total**	41	28	69
	Sensitivity	*Specificity*	
	90.24 %	85.71%	
	(76.87% to 97.28%)	(67.33% to 95.97%)	

Supplementary Table 1. Contingency table for ctDNA assay. The specificity and sensitivity of the ctDNA qPCR assay was calculated using data from Supplementary Table 2. See Materials and Methods for definitions of true positive, true negative, false positive and false negative ctDNA results.

Supplementary Table 2 Muhanna *et al.*

Table S2. Description of data used in Table S1

					Pre-Injection	
**Rabbit**	**True Positive**	**True Negative**	**False Positive**	**False Negative**	**True Negative**	**False Positive**
**1**	4	2				
**2**	3	2		1		
**3**	4	2				
**4**	3+1*			1	1	
**6**	8	1		1	1	
**5**	4+1*				1	
**7**	1	6	1	1	1	
**8**	8	1			1	
**9**					1	
**A**					1	
**B**					1	
**C**						1
**D**					1	
**E**					1	
**F**						1
**G**						1
**Sub-Total**	37	14	1	4	10	3
**Total**	**37** *True Positive*	**24** *True Negative*	**4** *False Positive*	**4** *False Negative*		

Supplementary Table 2. Description of data used in Table S1. The number of true positve, true negative, false positive, and false negative ctDNA results are shown for each of the rabbits injected with the VX2 cell lines in the study. The number of true negative and false positive ctDNA results are shown (gray box) for pre-injection blood from rabbits 4-9 and for seven additional uninjected rabbits designated A-G. Asterisks denote instances where ctDNA was detected for non-detectable tumour that subsequently developed.

Supplementary Figure 1 Muhanna *et al.*

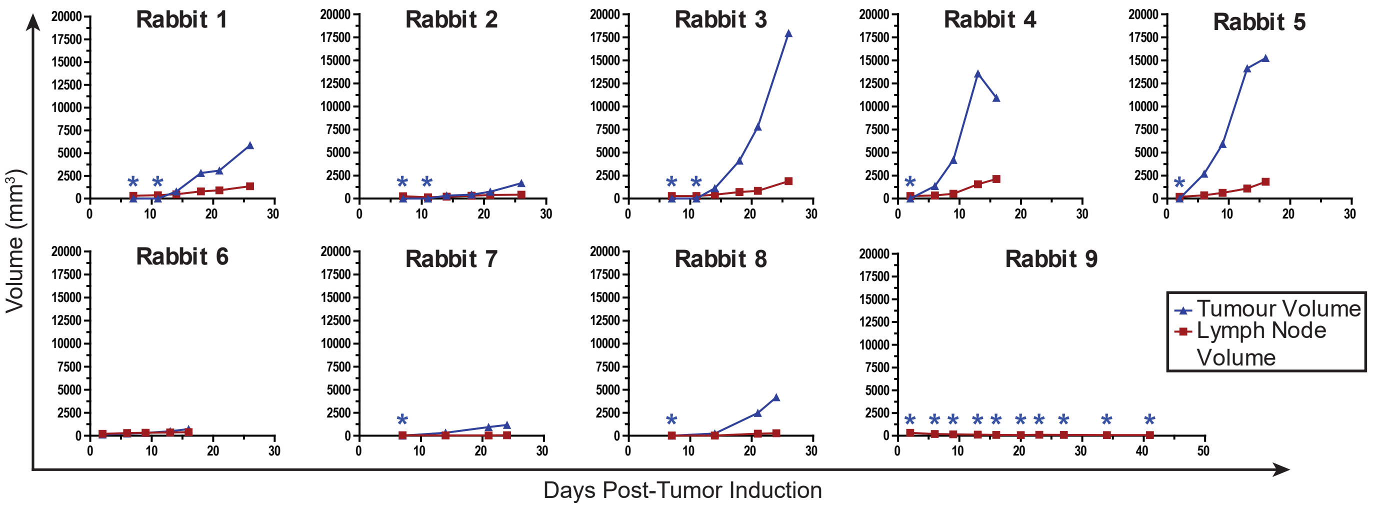

Supplemenary Figure 1: Volume of primary tumour (blue triangles) and lymph node (red squares) vs. time as measured by CT for all rabbits in this study. Asterisks denote non-detectable tumour size.

Supplementary Figure 2 Muhanna *et al.*

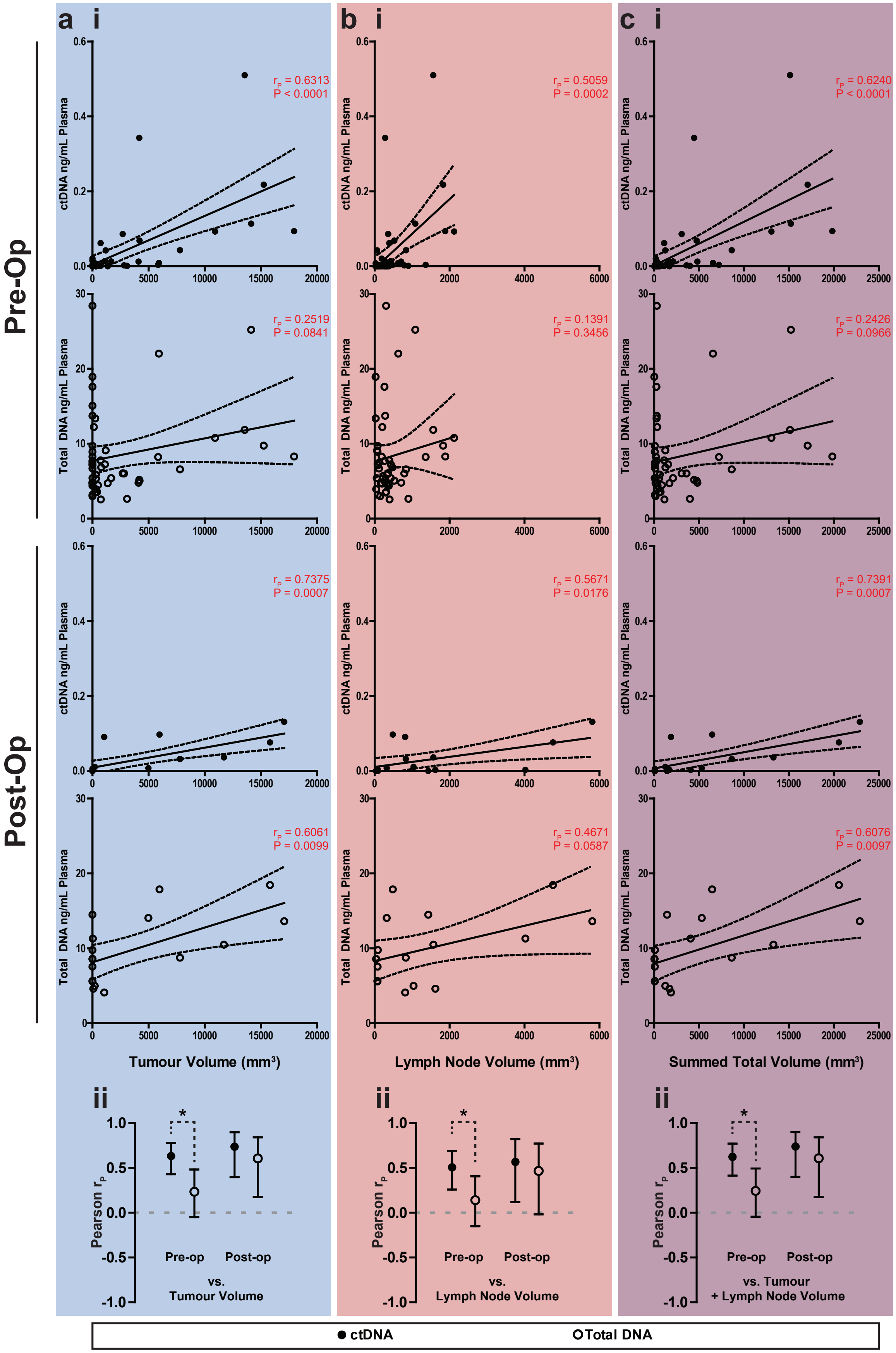

Supplementary Figure 2: Correlation of ctDNA or total plasma DNA vs. tumour volume (a), lymph node volume (b) or tumour + lymph node volume (c) in both the pre-operative and post-operative setting, as indicated. For each comparison, individual scatter plots with linear regression trend lines (solid) and 95% confidence intervals (dashed) are shown in (i). Summary plots of correlation coefficients (Pearson r, mean ±95% confidence interval) are shown in (ii). Asterisks indicate P < 0.05.

Supplementary Figure 3 Muhanna *et al.*

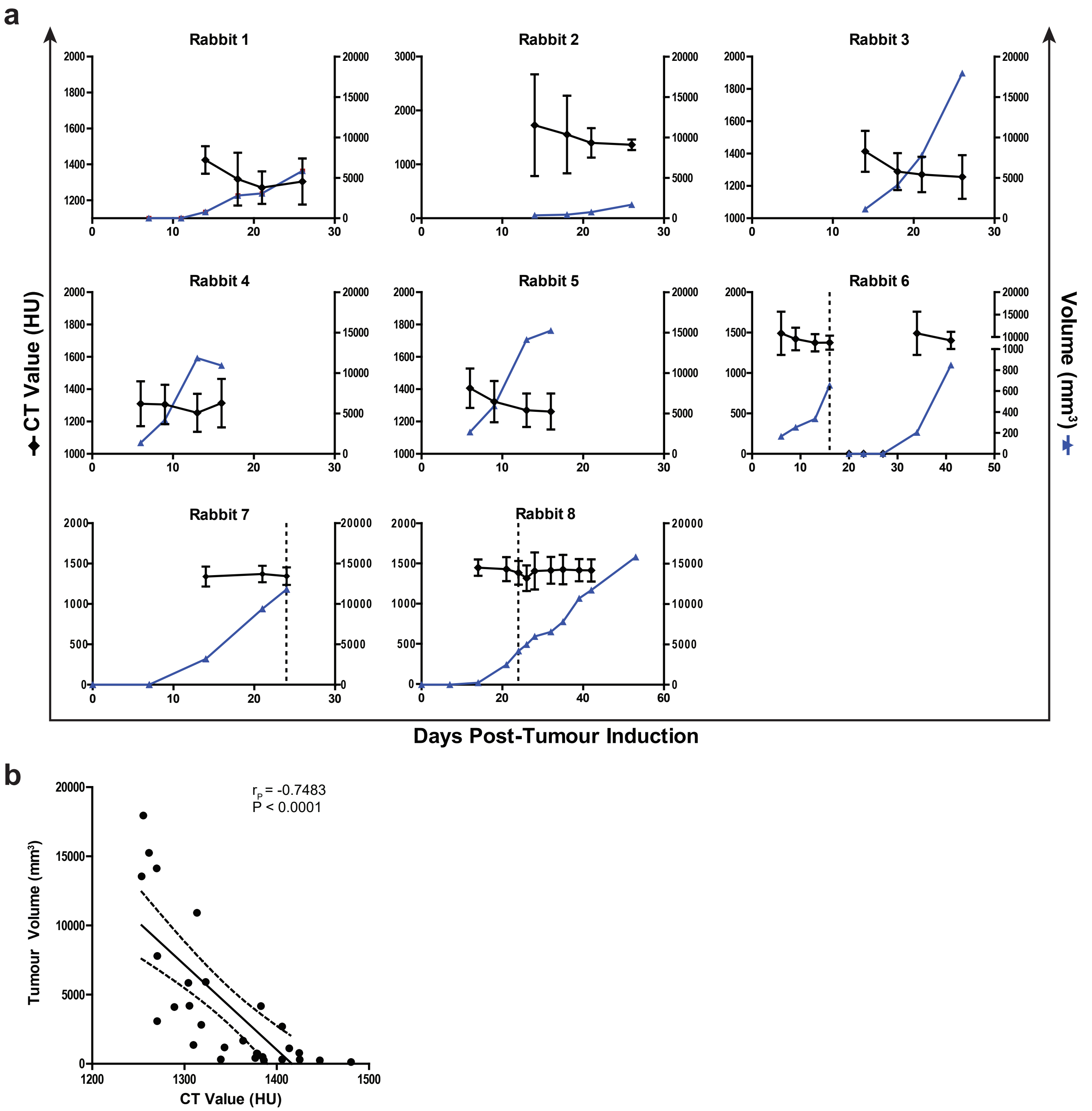

Supplementary Figure 3: (a) Correlation between average CT values (black) (Hounsfield Units, HU) and tumour volume (blue) of each tumour. Dotted vertical line represents the day of tumour resection surgery for Rabbits 6, 7, and 8. Error bars represent SD. (b) Correlation between average CT values (Hounsfield Units, HU) and tumour volume for all tumours in the study prior to operation. Linear regression trend line (solid) and 95% confidence intervals (dashed) are shown.
